# Is the right to health compatible with sustainability?

**DOI:** 10.7189/jogh.05.010301

**Published:** 2015-06

**Authors:** Daniel D Reidpath, Sofia Gruskin, Pascale Allotey

**Affiliations:** 1 Global Public Health, School of Medicine and Health Science, Monash University, Selangor, Malaysia; 2 Program on Global Health and Human Rights, Institute for Global Health, University of Southern California, Los Angeles, CA, USA

One of the fundamental human rights is the right of every individual to the enjoyment of the highest attainable standard of health, which we simplify to ‘HASH’ [1]. The HASH right was first articulated in the preamble to the WHO Constitution (1946) [2]. It appears in Article 12 of the International Covenant on Economic, Social and Cultural Rights (ICESCR) (1966) [3]; and occurs in various human rights treaties (cf [4]).

Fully realizing all human rights immediately and completely is impossible; and that is why the right to HASH is supported by the notion of progressive realisation (cf; ICESCR Article 2(1)[3][3]) [5]. That is, States have an obligation to take steps towards the progressive realisation of the right, with the result that over a period of time HASH, ideally, would be fully realised for each individual in the world [3]. Furthermore, economically developed states have an obligation which extends beyond their borders to progressively support less well developed states in achieving the vision [6].

In the recent manifesto *From public to planetary health*, Horton and colleagues declared their support for the HASH right [7] They went on to declare, *inter alia* that “our patterns of overconsumption are unsustainable and will ultimately cause the collapse of our civilisation”, that “the idea of unconstrained progress is a dangerous human illusion”, and that “we must conserve, sustain, and make resilient the planetary and human systems on which health depends by giving priority to the well being of all” [7]. There are, however, some fundamental public health challenges and trade–offs that need to be confronted for both HASH and sustainability agendas to be compatible. The trade–offs arise because, at a population level, the highest attainable standard of health is a standard that is achieved (or progressively realised) through unsustainable levels of consumption.

In 2013 a reasonable benchmark for the average highest attainable standard of health was 83 years of life; ie, the life expectancy in France, Iceland, Italy, Japan, and Switzerland (HASH–83) [8]. An individual’s HASH point, (ie, their individual right to the highest attainable standard of health) may actually be higher or lower than the population average; due, for example, to genetic (dis–)advantages. HASH–83, thus, potentially provides a policy benchmark for population performance, but it does not detract from a particular individual’s legal right to their (unknowable) true HASH. Therefore, and in the absence of specific individual data about adverse social determinants, favourable genes, and adverse pre–existing health states, the best guess for any randomly selected individual from a population (even a population with a life expectancy of 50 years) is that the selected individual has a progressively realisable right to HASH–83.

While we await the progressive realisation of an average HASH–83, we can expect that the current trend in life expectancy will actually increase, as it has been steadily doing over the past century. Here in lies the trade–off between the individual right to HASH and sustainability. The link between resource utilisation and health is well established. Nations with the highest GDP per capita and the highest levels of resource utilisation are the countries with populations achieving the highest standards of health, and populations increasing the HASH point [9].

The *Ecological Footprint* indicator was developed in the late 1990s to create a mechanism for measuring the sustainability of human use of the environment using uniform, globally available data [10]. Country level data allows a comparison of the per capita ecological footprint of each country in the common unit of global hectares of available land per capita (GHa/capita) [11]. Based on this indicator, the estimated sustainable footprint is about 1.8 GHa/capita [12]. The global average GHa/capita is about 2.7; ie, we ‘overshoot’ the sustainability threshold by 50%. Some countries have a considerably higher GHa/capita than others and currently “... about 84% of the world population lives in countries that run growing ecological deficits” (p.1) [13].

**Figure 1:**
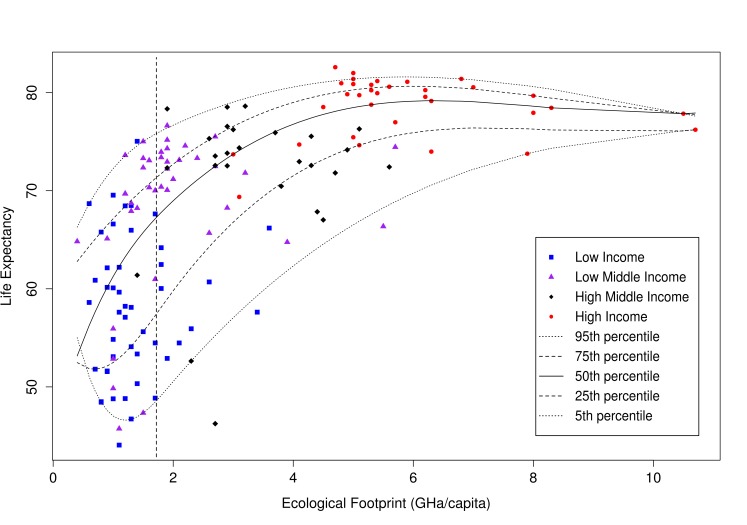
The relationship between the per capita ecological footprint and average life expectancy in 147 countries. GHa/capita – global hectares of available land per capita.

With the latest available ecological footprint data (2008) and life expectancy data from the World Bank for the same year [14], we charted the relationship between the per capita ecological footprint and average life expectancy in 147 countries (**Figure 1**). The relationship is plotted with a nonlinear quantile regression model at the 5^th^, 25^th^, 50^th^ (median), 75^th^ and 95^th^ percentiles. The broken vertical line is the sustainability threshold.

Countries’ average life expectancies rapidly increase with an increasing ecological footprint, and plateau (or perhaps decline slightly) after a GHa/capita of about 6. The median regression curve (solid black line), suggest a sustainable average life expectancy of 68 years, The countries with the highest average life expectancies (>80) are all high income countries, with a mean ecological footprint of 5.5 GHa/capita or three times the sustainability threshold.

These data suggest that, on average, the enjoyment of the highest attainable standard of health is most likely to be achieved by those living in the wealthiest countries and relies on the exploitation of the resources of the global commons. Unfortunately, at least some of the ecological footprint of high income countries is in fact transferred back to populations in low income countries in the form of technology [15], suggesting that the sustainable life expectancy may be even lower than 68 years.

Some countries achieve extremely good standards of health with much lower levels of resource utilisation and much lower levels of national wealth than others. This is shown in **Figure 1** by several of the low–middle income countries. They however still have populations for whom, on average, the HASH–83 has not been realised, and improvements in health will continue to rely on increasing consumption, fuelled by an increasing global population primarily in the poorest and on average least healthy countries.

In 2013 there were 7.2 billion people occupying the world [16]. On current projections the world’s population will increase by one third over the next 35 years (2050), giving rise to a total population of 9.6 billion people – and 10.9 billion by 2100 [16]. Each one of these people has a fundamental right to the progressive realisation of the HASH – at least HASH–83.

Let us accept as hyperbole the idea that everyone will achieve their fundamental human right to HASH. Equally, let us accept that the world’s population is likely to increase by one third by 2050. With 7 billion people already striving to be better off, we have failed to curtail our destruction of the planet’s rainforests [17]. We have failed to preserve the natural fish stocks in our oceans [18,19]. We have seen rates of species extinction in our life time which are associated with major planetary catastrophes [20]. Notwithstanding international commitments to reduce greenhouse gas production, the *rate* of production continues to increase [21,22]. Now add another 2.4 billion people. On very basic measures of sustainability we completely fail.

Those 2.4 billion extra people – the total population of the world a century earlier (1950) – will need food, shelter and health care along with their 7.2 billion companions. They will consume, and if they are like the people of the last quarter of a century, they will aspire to consume more than they do; and they will aspire to greater health than they currently have. We face the tragedy of the commons in which the commons we share is the entire planet. Growth in absolute consumption is not an unreasonable guess and does not auger well for human life on this planet, or for the life of many plant and animal species.

**Figure:**
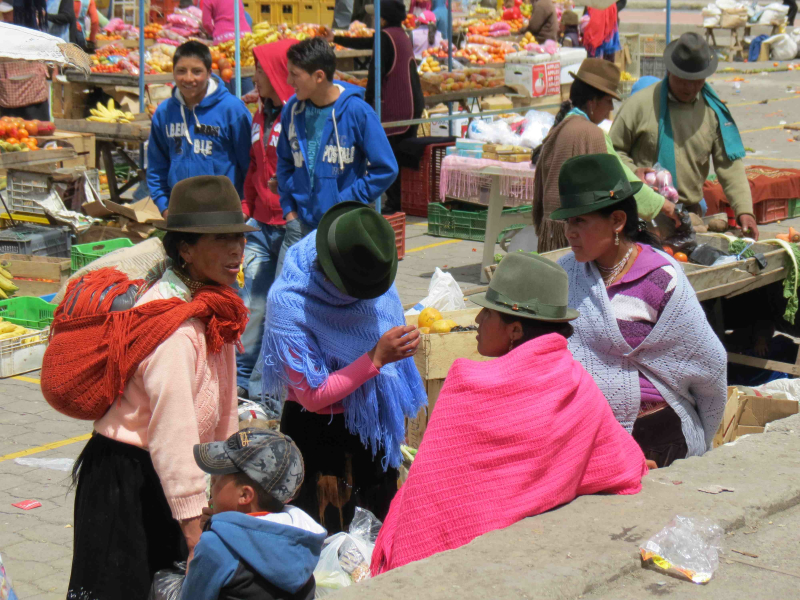
Photo: Courtesy of A. G. Klei, personal collection

The modern origins of the fundamental human right to the highest attainable standard of health was stirred by an optimistic vision of human progress and envisioned at a time in which global economic growth was seen as the way to achieve a just world. The reality today is markedly different. A future just world, a fair world, will be achieved not simply by improving the lot of the worst off alone, but simultaneously reducing the position of the best off and actively transferring benefits to the worst off. That is a bitter pill to swallow, made all the harder by the rhetorical assurances of the last quarter of a century that solutions lie in economic growth. Almost without exception, governments around the world are promising their populations that tomorrow (or perhaps the day after that) they will be healthier and wealthier. This is simply untrue. Unless we can reconcile ourselves to a life of (on average) fewer, but hopefully more dignified and rewarding years, and a life of less consumption but greater meaning, then we may lose the opportunity to choose our destiny at all.

The ideas behind *planetary health manifesto* are crucial – a “call to arms” [7]. However without confronting the critical compromises required to realise sustainable public and planetary health, it will remain a manifesto of good intentions. Our fundamental individual right to the highest attainable standard of health may need to focus explicitly on the quality and not necessarily the quantity of that life. At best we can claim a fundamental human right to the highest *sustainable* standard of health – and an obligation to take no more.
